# In This Issue

**Published:** 2008

## NEUROBIOLOGY OF ALCOHOL DEPENDENCE

Alcohol dependence is a debilitating disease that worsens over time, and chronic alcohol exposure causes changes in brain circuits that control motivational processes, including arousal, reward, and stress. Researchers are combining a plethora of approaches to identify and track the critical neural circuits that mediate positive and negative reinforcement, contribute to the transition from alcohol use and abuse to dependence, and underlie characteristics of dependence such as sensitization, tolerance, and withdrawal. In this article, Drs. Nicholas W. Gilpin and George F. Koob review the roles that brain circuits and neurotransmitter systems involved in reward and stress play at various stages of alcohol dependence. A better understanding of these systems may aid researchers in developing new treatment strategies. (pp. 185–195)

## COMMUNICATION NETWORKS IN THE BRAIN

Alcohol can affect numerous neurotransmitter, neurotrophin, and steroid hormone systems in the brain to produce acute intoxication, as well as neuroadaptations that contribute to tolerance and dependence. This article by Dr. David M. Lovinger reviews the chemical nature, neuronal actions, receptor subtypes, and therapeutic roles of several neurotransmitters, neurotrophins, and hormones. It focuses on neurotransmitters with important roles in acute and chronic alcohol effects on the brain, such as those that contribute to intoxication, tolerance, dependence, and neurotoxicity, as well as maintain alcohol drinking and addiction. (pp. 196–214)

## TRANSLATIONAL STUDIES OF ALCOHOLISM

Alcohol research, particularly research on alcohol's affect on the brain, has benefited from translational research—that is, research using both human subjects and animal models. In this article by Drs. Natalie M. Zahr and Edith V. Sullivan, the authors describe how studies in humans and animal models provide support for the involvement of specific brain structures over the course of alcohol addiction. The authors examine how translational research has helped to further theories in alcohol research relating different structures and circuitry in the brain to the rewarding (or reinforcing) effects of alcohol, the formation of habitual drinking and dependence, and the neuropathology that results from long-term drinking. (pp. 215–230)

**SPECIAL SECTION: TECHNOLOGIES FROM THE FIELD**

**Strategies to Study the Neuroscience of Alcoholism: Introduction**

Scientists are using a wide variety of strategies to learn about how alcohol affects the brain. This Special Section, edited by Drs. Robert Hitzemann and Denesa Oberbeck, provides a “top-down” approach to the latest strategies from the field, from examination of the whole brain, to specific brain regions, cells, molecules, and gene products. (pp. 231–232)

**Positron Emission Tomography As a Tool for Studying Alcohol Abuse**

In this article, Drs. Panayotis K. Thanos, Gene-Jack Wang, and Nora D. Volkow describe how positron emission tomography (PET)—a highly sensitive method that measures brain function in the human or animal body—is a potential tool in studying both alcohol’s effects on the brain and its effect on the brain’s neurochemistry. (pp. 233–237)

**From Event-Related Potential to Oscillations**

The brain’s electrical activity in humans typically has been measured using ongoing electroencephalograms (EEG) or by recording certain brain waves (i.e., event-related potentials) while the subject is performing a sensory or cognitive task. However, as Drs. Madhavi Rangaswamy and Bernice Porjesz describe in this article, changes in the dynamics of ongoing EEG rhythms, also known as event-related oscillations, may provide a means to better understand the network dynamics of brain functions and may serve as markers of alcoholism risk. (pp. 238–242)

**The Use of Magnetic Resonance Spectroscopy and Magnetic Resonance Imaging in Alcohol Research**

In this article, Drs. Bonnie J. Nagel and Christopher D. Kroenke review the recent emergence of magnetic resonance (MR)-based neuroimaging techniques, which have dramatically improved researchers’ ability to understand the neuropathology of alcoholism. These techniques have allowed researchers to monitor alcohol levels in the brain, identify alcohol-induced structural changes in the brain, and study the impact of alcohol on brain function. (pp. 243–246)

**Magnetic Resonance Imaging Approaches for Studying Alcoholism Using Mouse Models**

Magnetic resonance imaging (MRI) technologies are used in many research areas, including alcohol research, to assess brain function in humans. However, much of alcohol research involves mouse models to collect extensive genetic and behavioral data related to alcohol consumption and its consequences. As Drs. Eilis A. Boudreau, Gang Chen, Xin Li, and Christopher D. Kroenke report, the use of MRI technologies in mouse models is challenging, and only recently have investigators developed ways to use these noninvasive imaging techniques in rodents. (pp. 247–248)

**Laser-Assisted Microdissection**

Analyzing alcohol’s effects on the brain sometimes requires researchers to isolate small numbers of cells from a specific brain region without including unwanted cells. In this article, Dr. Denesa Oberbeck discusses the principles and uses of laser-assisted microdissection (LMD) as an approach to solving this problem. (pp. 249–250)

**Proteomic Solutions for Analytical Challenges Associated With Alcohol Research**

This article by Drs. Michael J. MacCoss and Christine C. Wu summarizes the challenges associated with proteomic analyses in animal models of alcohol dependence. The authors describe how a combination of label-free, discovery-based, and targeted quantitative proteomics strategies may help researchers overcome the challenges involved in studying the numerous proteins derived from animal models. (pp. 251–255)

**On the Use of Short-Interfering RNA to Study Alcohol-Related Genes**

One way to determine the contribution of individual genes to the development of complex traits and behaviors, such as alcohol consumption, is to inactivate a specific gene. In this article, Mr. Christopher A. Adams and Dr. W. Michael Zawada explore the use of small, artificially generated molecules called short-interfering RNAs (siRNAs) that allow researchers to selectively reduce or eliminate the expression of individual genes. (pp. 256–258)

**Viral Delivery of Small-Hairpin RNAs for Reducing Gene Expression in the Rodent Brain**

Although using short-interfering RNAs can effectively disrupt the expression of specific genes important in alcohol research, these effects are transient. As described by Drs. Amy W. Lasek and Ulrike Heberlein, an alternative strategy to ensure long-term expression of another type of interfering RNA molecule—small-hairpin RNAs (shRNAs)—is to introduce these RNA molecules into their target cells using viral delivery systems. (pp. 259–260)

**Quantitative Trait Locus Analysis**

Many alcohol-related behaviors, such as sensitivity to alcohol’s effects, are quantitative traits—characteristics that differ in the extent to which a person possesses them and which generally are influenced by more than one gene. In this article, Drs. Robert Hitzemann, John K. Belknap, and Shannon K. McWeeney describe how mapping of the genetic sequences related to these traits (i.e., of quantitative trait loci [QTL]) has become important in determining the genetic basis of such complex behaviors. (pp. 261–265)

**Interval-Specific Congenic Animals for High-Resolution Quantitative Trait Loci Mapping**

Mapping of quantitative trait loci (QTL) often is not specific enough to identify the gene(s) of interest. According to Ms. Deaunne L. Denmark, Ms. Lauren C. Milner, and Dr. Kari J. Buck, the use of interval-specific congenic (ISC) animals, which share all their genetic information except at one site, can improve the resolution of QTL mapping. (pp. 266–269)

**Single-Nucleotide Polymorphism Masking**

When trying to detect relevant genetic differences in gene expression between varying animal strains used in alcohol research, scientists may be hindered by the large number of small DNA variations (i.e, single-nucleotide polymorphisms [SNPs] naturally found in the animals’ DNA). As Ms. Nicole A.R. Walter, Dr. Shannon K. McWeeney, Ms. Sandra T. Peters, Dr. John K. Belknap, Dr. Robert Hitzemann, and Dr. Kari J. Buck explain, a strategy called SNP masking may be a valuable and feasible approach that may ameliorate these problems. (pp. 270–271)

**Expression Quantitative Trait Loci and the PhenoGen Database**

High-throughput screening technologies (i.e., microarray technologies) often identify large numbers of candidate genes that may be associated with a certain phenotype, such as alcohol dependence, and selecting the most likely ones is a difficult challenge. In this article, Dr. Laura Saba, Dr. Paula L. Hoffman, Ms. Cheryl Hornbaker, Dr. Sanjiv V. Bhave, and Dr. Boris Tabakoff suggest a strategy to narrow down the choices and present a tool (i.e., the PhenoGen Web site) that offers both the data and tools necessary for such analyses. (pp. 272–274)

**Integrative Genetic Analysis of Alcohol Dependence Using the GeneNetwork Web Resources**

One challenge in identifying genes related to alcoholism is to integrate data obtained at different levels and using different models in order to understand how genetic variants can cause differences in the risk of alcohol dependence. In this article, Drs. Robert W. Williams and Lu Lu introduce the GeneNetwork Web site as a useful resource for identifying specific genes that may contribute to alcohol-related phenotypes. (pp. 275–277)

